# Case Report: ST-Elevation Myocardial Infarction Secondary to Acute Atherothrombotic Occlusion Treated With No Stent Strategy

**DOI:** 10.3389/fcvm.2022.834676

**Published:** 2022-02-25

**Authors:** Rahul Dhawan, Saurabhi Samant, Ganesh Gajanan, Yiannis S. Chatzizisis

**Affiliations:** ^1^Cardiovascular Division, Mayo Clinic, Rochester, MN, United States; ^2^Cardiovascular Division, University of Nebraska Medical Center, Omaha, NE, United States

**Keywords:** STEMI, optical coherence tomography, intravascular imaging, acute coronary syndrome, plaque erosion, stent, percutaneous coronary intervention, case report

## Abstract

**Background:**

Intravascular imaging plays a vital role in the pathophysiology-based diagnosis and treatment of patients with ST-elevation myocardial infarction (STEMI). We present a case of STEMI due to plaque erosion, which was managed with a no stent approach.

**Case Summary:**

A 43-year-old female with a history of tobacco abuse presented with an anterior STEMI. Coronary angiography revealed acute thrombotic occlusion of the left anterior descending artery with spontaneous recanalization. Intravascular imaging with optical coherence tomography (OCT) demonstrated plaque erosion as the underlying etiology for the acute thrombotic occlusion. A no stent strategy with aspiration thrombectomy and dual antiplatelet therapy was used to manage the patient given that there was no evidence of plaque rupture. Repeat coronary imaging was done at 2 months to assess the status of the lesion.

**Conclusion:**

A 43-year-old female with STEMI due to plaque erosion was successfully managed only by thrombus aspiration and not by angioplasty and stent placement. Individualized treatment approaches in patients with acute coronary syndromes, can not only achieve optimal management goals but also avoid unnecessary complications associated with interventions. This case illustrates how intracoronary imaging and pathophysiology-guided treatment can dramatically change management. In this young patient, STEMI was managed purely by thrombus aspiration. Intravascular imaging obviated the need for stent placement possibly preventing stent-related complications including restenosis and thrombosis.

## Introduction

ST-elevation myocardial infarction (STEMI) can occur due to unstable coronary plaque rupture or plaque erosion. One-fourth of STEMI cases occur secondary to plaque erosion (1). The risk factors for plaque erosion include female gender, age <40, current tobacco abuse, and absence of multi-vessel disease (2). Intravascular imaging [i.e., intravascular ultrasound or optical coherence tomography (OCT)] is critical to diagnose the pathophysiology of STEMI and thereby help guide individualized treatment plan for patients. Currently, revascularization with coronary stent placement in patients presenting with STEMI is the mainstay of patient management, irrespective of the underlying pathophysiology. Early clinical studies have demonstrated promising results with a no stent management of acute coronary syndrome caused by plaque erosion (3). Currently, there is a lack of robust data from randomized clinical trials which support a no stent approach. We present a case of a patient with STEMI secondary to plaque erosion by OCT which was successfully managed without stent placement. The case report highlights the importance of intravascular imaging to determine the underlying pathology and assist with pathophysiology guided management.

## Case Presentation

A 43-year-old woman presented with dull retrosternal chest pain associated with diaphoresis for 5 h. She was in acute distress secondary to the pain. On admission, her heart rate was 109 beats per minute and her blood pressure was 160/100 mm Hg. Her oxygen saturation was 98% on room air. Further examination revealed regular cardiac rhythm, normal breath sounds, and absence of murmurs, jugular venous distension, or peripheral edema. She reported active tobacco abuse (30 pack-years smoking history), nulliparity, and no contraceptive medication use. The differential diagnosis included acute coronary syndrome (ACS), spontaneous coronary artery dissection, esophageal spasm, gastro-esophageal reflux disorder, pulmonary embolism, and musculoskeletal pain.

**Figure 1:**
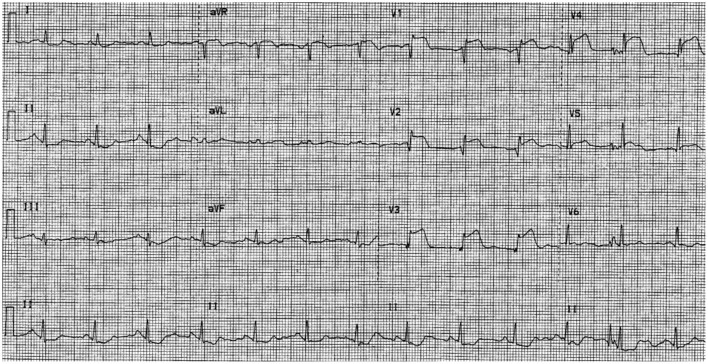
Twelve-lead ECG showing anterior wall ST-elevation myocardial infarction (ST elevations in leads V1–V4 with reciprocal changes in inferior leads).

A 12-lead ECG showed sinus rhythm with ST elevations > 2 mm in leads V1–V4 (Figure 1) with reciprocal changes in inferior leads, which led to a diagnosis of anterior STEMI. The chest X-ray showed a normal-sized heart with no consolidation or abnormality noted in the lungs.

**Figure 2:**
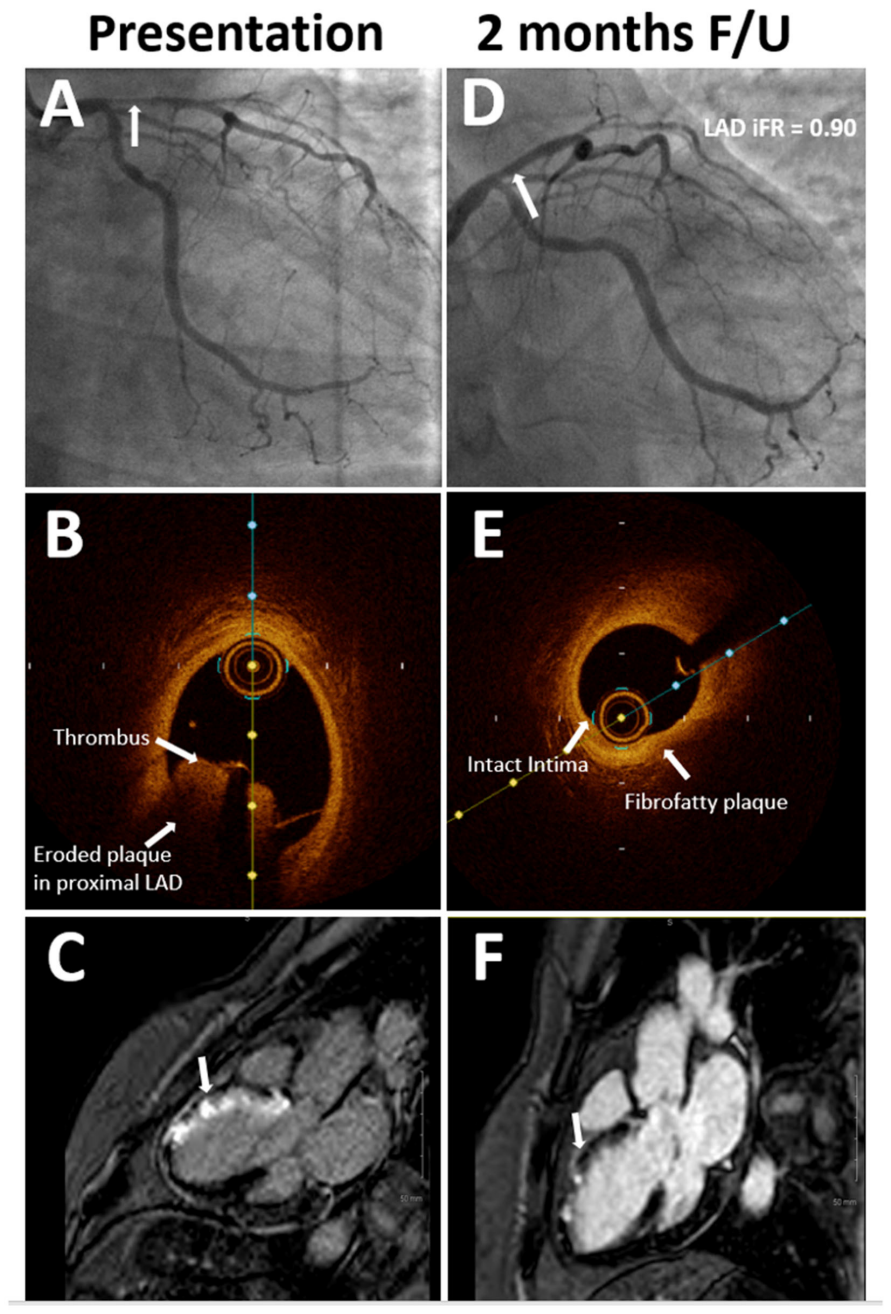
**(A)** Coronary angiography at presentation showing 80% stenosis (arrow) in proximal left anterior descending (LAD) with TIMI flow grade 2. **(B)** Optical coherence tomography (OCT) imaging at presentation showing significant thrombotic burden (arrow) and eroded plaque in the LAD. **(C)** Late gadolinium enhancement on cardiac magnetic resonance (CMR) imaging at presentation (arrow) consistent with LAD territory infarct. **(D)** Coronary angiography at 2-month follow-up showing non-obstructive stenosis (arrow) in proximal LAD. **(E)** OCT imaging at 2-month follow-up shows stable plaque in proximal LAD. **(F)** Non-transmural infarct was seen on the follow-up CMR imaging, which was done 6 weeks after the first presentation.

The patient was loaded with aspirin and ticagrelor, and emergently taken to the cardiac catheterization lab. Coronary angiography showed acute thrombotic occlusion of the proximal left anterior descending artery (LAD) with spontaneous recanalization and 80% stenosis, with thrombolysis in myocardial infarction (TIMI) flow grade 2 distally (Figure 2A). Given the significant thrombus burden, we proceeded with aspiration thrombectomy which established TIMI flow grade 3. Following thrombectomy, optical coherence tomography (OCT) of the LAD was performed to evaluate the underlying etiology for the acute coronary event. OCT revealed a significant residual thrombotic burden overlying an eroded, non-obstructive plaque (Figure 2B). After additional rounds of thrombectomy, repeat OCT showed plaque erosion with no evidence of plaque rupture. A final coronary angiography revealed 50% residual stenosis. Stenting was deferred, given the non-obstructive nature of the underlying plaque, absence of plaque rupture, restoration of TIMI flow grade 3, and improvement of symptoms and resolution of the ST elevation on ECG (Electrocardiogram). An Eptifibatide bolus of 180 mcg/kg was administered, followed by a 2 mcg/kg/min infusion for 18 h. On day 3 of admission, cardiac MRI (CMR) showed a left ventricular ejection fraction (LVEF) of 50% and myocardial changes consistent with the subacute stage of medium-sized infarction in the LAD territory. Specifically, we noted late gadolinium enhancement involving the basal to mid anteroseptal, distal septal, and distal anterior walls, consistent with a transmural infarction (Figure 2C). The patient was discharged on dual antiplatelet therapy and high-intensity statin (atorvastatin 80 mg daily). Smoking cessation counseling was done, nicotine patches were prescribed, and the patient was enrolled for cardiac rehabilitation therapy.

At 2 months follow-up, the patient was asymptomatic. Repeat angiogram showed a non-obstructive lesion in the proximal LAD (Figure 2D). Functional assessment with instantaneous wave-free ratio (iFR) confirmed that the intermediate stenosis was not hemodynamically significant (iFR=0.90). OCT showed a stable, non-obstructive, thick cap fibro-fatty plaque in the proximal LAD with no evidence of thrombus (Figure 2E). Repeat CMR showed remarkable recovery with improvement in the LVEF to 58% and the late gadolinium enhancement pattern was now consistent with a non-transmural infarction of the mid anteroseptal (50% involvement), distal anterior, and distal septal segments (50–75% involvement) (Figure 2F). Her low-density lipoprotein at the time of presentation was 79 mg/dl which improved to 54 mg/dl on a high-intensity statin. On her most recent follow-up, 27 months after the initial presentation, she continued to do well. She had successfully quit tobacco with counseling and temporary nicotine replacement therapy, and her last cigarette use was on the day of her myocardial infarction (Table 1). We plan to continue to treat her with dual antiplatelet therapy for as long as she can tolerate it without bleeding issues and consider anatomic or functional assessment (preferably a non-invasive study with coronary computed tomography angiography (CCTA) or stress testing) only if she were to have symptoms concerning for angina.

**Table 1:** Timeline.

Presentation	•Retrosternal chest pain for 5 h •EKG showed STEMI •Loaded with aspirin and ticagrelor •Shifted to the catheterization lab
Cath lab	•Cardiac catheterization showed acute thrombotic occlusion with spontaneous recanalization and residual 80% stenosis in LAD with TIMI flow grade 2 • Performed aspiration thrombectomy • OCT of LAD showed plaque erosion and a non-obstructive plaque • Additional rounds of aspiration thrombectomy were performed • 50% residual LAD stenosis on angiography and TIMI flow grade 3
Post-Procedure	•Eptifibatide drip was administered over 18 h
Day 3	•Left ventricular ejection fraction was 50% on cardiac magnetic resonance imaging • Patient was discharged on dual anti-platelet therapy and statin
2 Months	•Stable non-obstructive CAD which was not hemodynamically significant by iFR on repeat cardiac catheterization

## Discussion

The proportion of ACS due to plaque erosion may be increasing due to the widespread use of medications, including statins (4). In this case, intravascular imaging played a vital role in identifying plaque erosion as the underlying pathophysiology of ACS. Given the findings of plaque erosion with an underlying non-obstructive plaque, we elected to manage this young patient using a no stent approach which would also avoid potential complications secondary to stent placement including perforation, in-stent restenosis, and thrombosis.

A no stent treatment approach is an evolving approach to treating patients with underlying plaque erosion. A prospective study evaluating this approach in ACS secondary to plaque erosion showed that 84% of patients got aspiration thrombectomy, and about 64% received a glycoprotein IIb/IIIa inhibitor. In addition, these patients also received optimal medical therapy including aspirin, ticagrelor, and high-intensity statin (5). One-year follow-up showed that 92.5% of patients had no major cardiovascular events (3). Other studies also elucidated successful results in patients with plaque erosion treated with a no stent approach (6, 7). Patients with plaque erosion treated with stents had a higher percentage of uncovered struts, lower neointimal area, and neointimal thickness at 6-month follow-up, which leads to unfavorable vascular healing (8). CMR imaging is recommended to visualize tissue and injury characterization in patients with STEMI. In our case, we assessed the short and long-term endpoints regarding infarct size and left ventricular ejection fraction with CMR imaging (9).

Initial studies have also identified five clinical and laboratory predictors of plaque erosion in patients with acute coronary syndrome: younger age, anterior ischemia, absence of diabetes mellitus, higher hemoglobin, and normal renal function (10). These parameters might be helpful for possible targeted therapy of plaque erosion leading to STEMI, although intravascular imaging continues to be the cornerstone for diagnosis and decision making. However, with the increasing adoption of CCTA, it is possible to identify high-risk patients early and treat them with aggressive medical therapy to prevent ACS. The major advantage of CCTA is its incredible ability to provide a detailed characterization of atherosclerotic plaque composition, and thereby help identify vulnerable plaques which are precursors of ACS (11).

Our case illustrates how intracoronary imaging and pathophysiology-guided treatment can dramatically change management: In our patient, STEMI was managed purely by thrombus aspiration and intravascular imaging obviated the need for stent placement and possibly prevented stent related complications including restenosis and thrombosis. Of note, this management plan was tailored to this young patient. The standard of care and current guidelines recommend stent placement in similar cases. However, increasing evidence, including data from recent randomized control trials (12) highlights the need for large-scale randomized clinical trials to achieve a paradigm shift toward pathophysiology-based treatment in acute coronary syndrome.

## Data Availability Statement

The original contributions presented in the study are included in the article, further inquiries can be directed to the corresponding author.

## Author Contributions

RD involved with the management of the patient and leading the write-up of the manuscript. SS made significant contributions to writing the manuscript and proofreading. GG made significant contributions to writing the manuscript, proofreading, and submitting. YC involved directly in treating the patient, mentored, and made suggestions in the preparation of the manuscript. All authors contributed to the article and approved the submitted version.

## Conflict of Interest

YC has speaker honoraria, advisory board fees and a research grant from Boston Scientific Inc. and a research grant from Medtronic Inc. The remaining authors declare that the research was conducted in the absence of any commercial or financial relationships that could be construed as a potential conflict of interest.

## Publisher's Note

All claims expressed in this article are solely those of the authors and do not necessarily represent those of their affiliated organizations, or those of the publisher, the editors and the reviewers. Any product that may be evaluated in this article, or claim that may be made by its manufacturer, is not guaranteed or endorsed by the publisher.

## Abbreviations

CMR: cardiac magnetic resonance imaging
LAD: left anterior descending artery
OCT: optical coherence tomography
TIMI: thrombolysis in myocardial infarction
STEMI: ST elevation myocardial infarction.
